# Protocol for the isotoxic intensity modulated radiotherapy (IMRT) in stage III non-small cell lung cancer (NSCLC): a feasibility study

**DOI:** 10.1136/bmjopen-2015-010457

**Published:** 2016-04-15

**Authors:** Kate Haslett, Kevin Franks, Gerard G Hanna, Susan Harden, Matthew Hatton, Stephen Harrow, Fiona McDonald, Linda Ashcroft, Sally Falk, Nicki Groom, Catherine Harris, Paula McCloskey, Philip Whitehurst, Neil Bayman, Corinne Faivre-Finn

**Affiliations:** 1 Radiotherapy Related Research, The Christie NHS Foundation Trust, Manchester, UK; 2 Leeds Cancer Centre, St. James's University Hospital, Leeds Teaching Hospitals NHS Trust, Leeds, UK; 3 Department of Clinical Oncology, Centre for Cancer Research and Cell Biology, Queen's University of Belfast, Belfast, UK; 4 Department of Clinical Oncology, Cambridge University Hospitals NHS Foundation Trust, Addenbrookes Hospital, Cambridge, UK; 5 Department of Clinical Oncology, Weston Park Hospital, Sheffield, UK; 6 Department of Clinical Oncology, Beatson West of Scotland Cancer Centre, Glasgow, UK; 7 Department of Radiotherapy, The Royal Marsden, NHS Foundation Trust, London, UK; 8 Manchester Academic Health Science Centre Trials Co-ordination Unit (MAHSC-CTU), The Christie NHS Foundation Trust, Manchester, UK; 9 Radiotherapy Trials Quality Assurance Team, Mount Vernon Hospital, Northwood, UK; 10 Christie Medical Physics and Engineering, The Christie NHS Foundation Trust, Manchester, UK; 11 Department of Clinical Oncology, Northern Ireland Cancer Centre, Belfast, UK; 12 Department of Clinical Oncology, The Christie NHS Foundation Trust, Manchester, UK; 13 The University of Manchester, Manchester Academic Health Science Centre, Institute of Cancer Sciences, Manchester Cancer Research Centre (MCRC), Manchester, UK

## Abstract

**Introduction:**

The majority of stage III patients with non-small cell lung cancer (NSCLC) are unsuitable for concurrent chemoradiotherapy, the non-surgical gold standard of care. As the alternative treatment options of sequential chemoradiotherapy and radiotherapy alone are associated with high local failure rates, various intensification strategies have been employed. There is evidence to suggest that altered fractionation using hyperfractionation, acceleration, dose escalation, and individualisation may be of benefit. The MAASTRO group have pioneered the concept of ‘isotoxic’ radiotherapy allowing for individualised dose escalation using hyperfractionated accelerated radiotherapy based on predefined normal tissue constraints. This study aims to evaluate whether delivering isotoxic radiotherapy using intensity modulated radiotherapy (IMRT) is achievable.

**Methods and analysis:**

Isotoxic IMRT is a multicentre feasibility study. From June 2014, a total of 35 patients from 7 UK centres, with a proven histological or cytological diagnosis of inoperable NSCLC, unsuitable for concurrent chemoradiotherapy will be recruited. A minimum of 2 cycles of induction chemotherapy is mandated before starting isotoxic radiotherapy. The dose of radiation will be increased until one or more of the organs at risk tolerance or the maximum dose of 79.2 Gy is reached. The primary end point is feasibility, with accrual rates, local control and overall survival our secondary end points. Patients will be followed up for 5 years.

**Ethics and dissemination:**

The study has received ethical approval (REC reference: 13/NW/0480) from the National Research Ethics Service (NRES) Committee North West—Greater Manchester South. The trial is conducted in accordance with the Declaration of Helsinki and Good Clinical Practice (GCP). The trial results will be published in a peer-reviewed journal and presented internationally.

**Trial registration number:**

NCT01836692; Pre-results.

**Strengths and limitations of this study**

- Radiotherapy delivered using advanced techniques, including intensity modulated radiotherapy (IMRT), 4D-CT and image-guided radiotherapy (IGRT) in all patients.
- Robust quality assurance (QA) programme.
- Multicentre study.
- Single arm study.
- Heterogeneity of the stage III patient group.

## Introduction

Lung cancer is the leading cause of cancer mortality worldwide^1^ with approximately 40 000 new cases diagnosed annually in the UK. Of these cases 34 000 will present with non-small cell lung cancer (NSCLC) and one-third (∼12 000) of patients with NSCLC will present with locally advanced (stage III) disease. The 5-year survival from lung cancer in the UK has changed little (from 3% to 8%) over the past 60 years, with progress lagging significantly behind other common cancers. The 5-year survival of stage III NSCLC with current standard treatment is approximately 10–15% highlighting a real urgency to improve outcome for these patients.

Radiotherapy (RT), alone or combined with chemotherapy, plays a major therapeutic role in the treatment of stage III NSCLC. Despite this, most patients still progress locally and at sites of distant spread. Concurrent chemoradiotherapy (chemotherapy and RT given at the same time; CTRT) is the standard of care in stage III NSCLC^2^ with median survival rates of approximately 21 months. However, the majority of patients are not suitable for this treatment based on poor performance status (PS) and comorbidities.^3^ In a recent UK national survey of CTRT practice, the majority of clinical oncologists estimated that <30% of patients with stage III NSCLC were suitable for concurrent CTRT.^4^ The alternative treatment offered to patients who are not suitable for concurrent CTRT is sequential CTRT (chemotherapy given prior to RT), but local control and survival rates are inferior compared with concurrent CTRT.^2^ As the majority of patients with stage III NSCLC cannot be treated concurrently, strategies to improve outcome in this large group of patients treated sequentially is vital.

Local control with current RT doses delivered with standard three-dimensional conformal RT (3D-CRT) is poor with local progression-free survival rates of about 30%. However, recent data has shown that improved local control in lung cancer can lead to improvement in survival.^2^ A meta-analysis of concurrent versus sequential CTRT in locally advanced NSCLC based on individual patient data demonstrated that although concurrent treatment decreases locoregional progression (HR=0.77; p=0.01); its effect is not different from that of sequential treatment on distant progression (HR=1.04; p=0.69).^2^ The decrease in locoregional progression translated into a significant survival benefit in favour of concurrent CTRT (HR, 0.84; 95% CI 0.74 to 0.95; p=0.004), with an absolute benefit of 5.7% at 3 years and 4.5% at 5 years.

We are now accelerating into an era of personalised medicine for the systemic treatment of NSCLC. However, to date, fixed doses of radiation are still delivered to patients, not taking into account volume of disease, stage of disease (IIIa vs IIIb) or anatomical location within the thorax. Stage III NSCLC is a heterogeneous disease and there is a need to move away from a ‘one-size-fits-all’ approach to more personalised radiation treatments. The concept of isotoxic RT was recently introduced allowing the radiation dose prescription to the tumour to be tailored based on normal tissue constraints.^5^ ^6^

A strategy to improve local control is to escalate the dose of radiation delivered to the tumour. Martel *et al*^7^ demonstrated a clear dose–response relationship in NSCLC, with 84 Gy using conventional fractionation required to achieve 50% probability of tumour control at 3 years. Subsequently we have learnt from the stereotactic body RT studies in NSCLC that biologically effective dose (BED) in excess of 100 Gy are necessary to achieve >90% local control rates.^8^

However, a recent Radiation Therapy Oncology Group (RTOG) phase III study that randomised patients between 60 Gy in 30 daily fractions and 74 Gy in 37 daily fractions has failed to demonstrate a survival advantage for the high-dose arm indicating that dose escalation using a conventional fractionation resulting in increased overall treatment time is not the way forward in this disease. The failure of the high-dose arm was likely multifactorial and in addition to prolonged overall treatment time may have resulted from a combination of poorer treatment delivery with less patients receiving concurrent chemotherapy, reduced compliance to RT, QA issues and unreported treatment toxicity.^9^ Following the presentation of RTOG 0617 a dose of 60 Gy biologically equivalent dose in 2 Gy fractions (EQD2) is considered to be standard in patients with stage III NSCLC.^10^

Accelerated hyperfractionation has been studied in an attempt to reduce the overall treatment time and counteract repopulation in lung cancer. In the national continuous hyperfractionated accelerated radiotherapy (CHART) study there was a staggering 24% reduction in the relative risk of death, which is equivalent to an absolute improvement in 2-year survival of 9% with hyperfractionated accelerated RT (54 Gy in 36 fractions over 12 days) compared with conventional RT (60 Gy in 30 fractions over 6 weeks).^11^ Despite CHART showing this significant benefit, it has not become standard practice in the UK. First, a large percentage of patients included had stage I–II disease (36%) who would now be considered for surgery or stereotactic body radiotherapy (SABR), and second, the control arm would not be considered current standard of care since chemotherapy was not delivered with RT (either sequentially or concurrently). Subsequently an individual patient data meta-analysis of 2000 patients from 10 trials, demonstrated that modified fractionation (acceleration, hyperfractionation or both) improves overall survival as compared with conventional fractionation in NSCLC resulting in an absolute benefit of 3% at 5 years.^12^

Intensity modulated radiotherapy (IMRT) modulates the intensity profile of the radiation delivered to the patient allowing improved targeting of the radiation dose. This technique allows a decrease in the mean lung dose (MLD), V20 (percentage volume of total lung receiving ≥20 Gy), and maximal spinal cord dose. As a result the dose delivered to the tumour can be escalated while keeping the dose to the normal tissue within tolerance.^13^ Although IMRT is becoming standard for the treatment of lung cancer in large international academic centres, in the UK implementation of IMRT is currently poor. In September 2010 Cancer Research UK reported that UK RT practice is ‘lagging behind’ with only 7% of patients receiving IMRT compared with 20% in Europe.

The MAASTRO group have pioneered the concept of ‘isotoxic RT’ allowing for individualised dose escalation using hyperfractionated accelerated RT based on predefined MLD and spinal cord dose in stage I–III patients.^6^ They have shown with 3D-CRT delivered two times a day over 4 weeks that increasing the radiation dose to prespecified normal tissue dose constraints could lead to increased tumour control probability with the same normal tissue complication probability. In the stage III group of patients the mean dose delivered was 61.2 Gy (ie, 72.2 Gy BED10 and 60.2 Gy EQD2), range 50.4–79.2 Gy (ie, 59.5 Gy BED10 and 49.6 Gy EQD2 –93.5 Gy BED10 and 77.9 Gy EQD2) and <10% of patients received the maximum dose as per protocol of 79.2 Gy in 39 fractions two times a day with 3D-CRT (ie, 95.3 Gy BED10, 79.4 Gy EQD2). The survival rates of stage III patients in this study, all of whom were treated with sequential CTRT, were comparable to the results expected with concurrent CTRT with acceptable acute and late toxicity. It is important to note that IMRT was not used in the MAASTRO study. Our in-house planning study, has confirmed the use of IMRT could allow further individual dose escalation in this group of patients, which may ultimately translate into improved survival.^14^

## Methods and analysis

The Isotoxic IMRT study is a non-blinded multicentre feasibility study. The study is sponsored by The Christie NHS Foundation Trust^*^ and coordinated by the Manchester Academic Health Science Centre Trial Co-ordination Unit (MAHSC-CTU) based at The Christie NHS Foundation Trust. Data management is undertaken by the MAHSC-CTU. The trial is registered on the clinicaltrials.gov database (NCT01836692) and jointly funded by Cancer Research UK's Clinical Trials Awards and Advisory Committee (CTAAC) and the British Lung Foundation (BLF). The study is included in the National Institute for Health Research (NIHR) Clinical Research Network portfolio (ID: 14937). The trial is conducted in accordance with the Declaration of Helsinki and Good Clinical Practice (GCP).

The primary research question is to evaluate the feasibility of delivering isotoxic RT using IMRT and hyperfractionated accelerated RT in patients with stage III NSCLC unsuitable for concurrent CTRT.

The secondary research questions are:

1. Estimate the feasibility of delivering lung isotoxic IMRT
2. Estimate the proportion of patients with acute grade 3+ non-haematological toxicity
3. Estimate late toxicity
4. Estimate local control and overall survival
5. Develop a robust QA process for lung IMRT

### Setting

In total, 35 patients with a histological or cytological proven diagnosis of NSCLC will be recruited from seven UK centres: Addenbrookes (Cambridge), Beatson (Glasgow), The Christie NHS Foundation Trust (Manchester), Royal Marsden (London), Northern Ireland Cancer Centre (Belfast), Weston Park (Sheffield), St James's Hospital (Leeds). The study started recruitment in June 2014.

**Figure 1:**
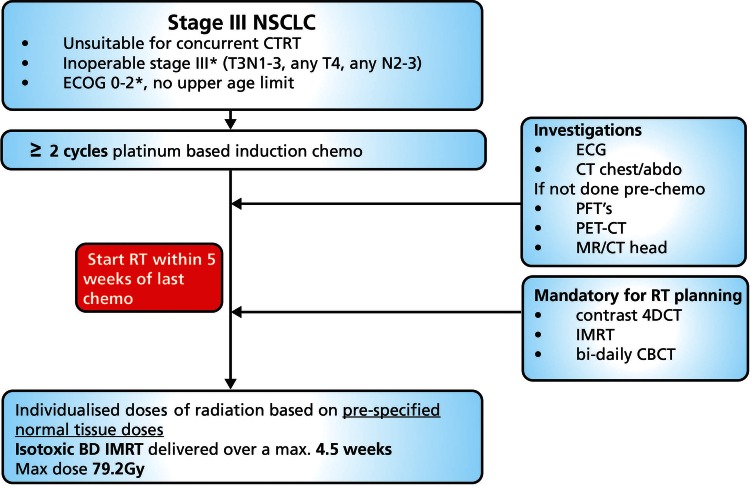
Trial schema. BD, two times a day; CBCT, Cone beam computer tomography; CTRT, chemoradiotherapy; ECOG, Eastern Cooperative Oncology Group; IMRT, intensity modulated radiotherapy; NSCLC, non-small cell lung cancer; PET, positron emission tomography; PTFs, pulmonary function tests; RT, radiotherapy.

Patients with stage III NSCLC, PS 0–2, not suitable for concurrent CTRT, will be treated with individualised doses of radiation based on prespecified normal tissue doses (spinal cord, brachial plexus, lung tissue, heart and great vessels/proximal bronchial tree table 1). The study flow diagram is shown in figure 1.

**Table 1:** Prespecified normal tissue doses (specified to a volume of 1 cc)

Organ at risk	Prespecified normal tissue doses
Brachial plexus	Maximum dose=EQD2≤66 Gy
Heart	Maximum dose=EQD2≤76 Gy Mean dose ≤46 Gy
Lung	Mean lung dose (lung—GTV) ≤20 Gy
Mediastinal envelope*	Maximum dose=EQD2≤76 Gy
Spinal canal PRV	Maximum dose=EQD2≤50 Gy

*Including: heart, proximal bronchial tree, trachea, oesophagus and the blood vessels in the upper mediastinum.

EQD2, equivalent dose in 2 Gy fractions; GTV, gross tumour volume; PRV, planning organ at risk volume.

### Participant screening and selection

Patients have been deemed inoperable by the lung multidisciplinary team (MDT) and suitable for sequential CTRT. Eligible patients are invited to participate and provided with a patient information sheet (see online supplementary appendix 1). Patients are only registered into the Isotoxic IMRT study once. Mandatory investigations prior to registration included: a Contrasted CT scan of the thorax and upper abdomen (within 4 weeks prior to registration), Contrast-enhanced CT (or MRI) brain scan (within 4 weeks prior to registration if patients have not had imaging of the brain prior to starting induction chemotherapy), fluorodeoxyglucose-positron emission tomography (FDG-PET) CT within 4 weeks prior to registration if patients have not had a PET-CT prior to starting induction chemotherapy and lung function tests.

10.1136/bmjopen-2015-010457.supp1 Supplementary appendix 1

### Inclusion criteria

- Histologically or cytologically confirmed NSCLC
- Inoperable stage III disease (T3N1-3, any T4, any N2-3) confirmed by PET scanning, mediastinoscopy or thoracoscopy
- Patients treated with at least two cycles of platinum-based induction chemotherapy and able to start RT within 5 weeks of the last cycle of chemotherapy
- Tumour judged inoperable by a lung MDT
- Age ≥18, no upper age limit
- PS—Eastern Cooperative Oncology Group (ECOG) scale 0–2. Patients with PS 2 whose general condition is explained by disease can be included at the discretion of the local investigator. Patients with PS 2 as a result of comorbid conditions will be excluded
- Patient considered suitable for radical RT
- Tumour that can be encompassed within a radical RT treatment volume (MLD expected to be ≤20 Gy).

### Exclusion criteria

- Patients suitable for standard concurrent CTRT
- Patients only suitable for radical RT alone

### Informed consent

Eligibility to participate is confirmed by a clinician prior to consent being taken. Patients are given at least 24 h to consider the patient information sheet and time to ask questions prior to written informed consent being taken by a trial doctor. The consent form can be viewed in online supplementary appendix 2.

10.1136/bmjopen-2015-010457.supp2 Supplementary appendix 2

### Registration

Registration into this study takes place after completion of chemotherapy. Once a patient is deemed eligible for the study and has consented to participate, the MAHSC-CTU will allocate a participant identification number by telephone and confirm enrolment by email.

### Standard care

In patients with inoperable stage III NSCLC who are unsuitable for concurrent CTRT, sequential CTRT is the standard of care. While on study participants will not co-enrol in other clinical trials offering therapeutic intervention. Patients can withdraw from the trial at any time without any effect on clinical care.

### RT intervention and planning

4D-CT scanning is mandatory to account for tumour motion during the breathing cycle. The use of intravenous contrast is mandated (patients with a medical contraindication should not be included in the study). The whole thorax (cricoid to L2) should be covered to allow dose volume histograms to be calculated for the lung, heart, spinal cord, brachial plexus, great vessels, proximal bronchial tree and the oesophagus. RT should be started within 3 weeks of 4D-CT planning scan date.

- The motion adapted gross tumour volume (GTV) is defined as identifiable tumour and involved lymph nodes.
- The clinical target volume (CTV) will encompass regions at risk of microscopic extension.
- The CTV comprises the GTV with a 5 mm margin of radiologically normal tissue in all directions.
- CTV to planning target volume (PTV) expansion takes into account patient set-up uncertainties. Tumour motion has been taken into account within the ‘motion-adapted GTV’.
- The PTV comprises the CTV with a 0.9 cm margin superiorly and inferiorly, and 0.7 cm margin laterally.
- Editing of the PTV is not permitted.

### Prescribed dose and fractionation

RT doses will be individually escalated until one or more of the organs at risk (OAR) tolerance or the maximum dose of 79.2 Gy is reached. At least 95% of the PTV should receive 90% (ideally 95%) of the prescribed dose and the mean dose to the CTV should be 100%. Hotspots should not exceed 107% of the prescribed dose within a 1 cc volume. IMRT will be delivered two times a day on consecutive weekdays, at 1.8 Gy per fraction, with a minimum 6 h interval between fractions. A maximum of 44 fractions will be delivered for the maximum dose of 79.2 Gy. The overall treatment time should include as few weekends as possible.

Prescribing the treatment using the mean dose to the PTV for lung tumours can be problematic. In certain situations low-density lung tissue at the edge of the PTV can lead to an exaggerated increase in the monitor units needed to achieve the necessary mean dose. For this reason it is suggested that the mean dose will be prescribed to the CTV.

### Dosimetry/dose specifications

Treatment planning will be with inverse planned IMRT. This optimisation must be performed by an experienced dosimetrist/physicist in lung planning.

The use of volumetric modulated arc therapy (VMAT)/RapidArc/tomotherapy/fixed-beam IMRT is allowed in this study. If fixed-beam IMRT is used, it is suggested that five or more fields should be used to avoid creating hot-spots in normal tissue and ensure optimal dose distribution. The use of these techniques facilitate the mandatory PTV coverage of D95 ≥90% (ideally 95%) of the prescribed dose and a 1 cc maximum <107%.

Patients are treated with individualised doses of radiation based on prespecified normal tissue constraints (spinal cord, brachial plexus, lung tissue, heart and great vessels/proximal bronchial tree). RT is delivered two times a day over a maximum period of 4.5 weeks using IMRT and the dose of radiation is increased until one or more of the OAR tolerance or the maximum dose of 79.2 Gy is reached.

### Follow-up

Patients are followed up for 5 years post-treatment (4 monthly in years 1 and 2, 6 monthly from years 2–5). A late toxicity assessment will be performed at each visit.

### Statistical considerations

This feasibility study will be two-stage (with early stopping rules) using the design of Bryant and Day with 85% power and 15% significance level for both completion and acute radiation pneumonitis rates. Using the design of Bryant and Day^15^ to combine an acceptable rate of 90% and unacceptable rate of 70% of patients receiving >60 Gy EQD2 and an acceptable rate of grade 3+ acute radiation pneumonitis of 8.5% and unacceptable rate of 22.5%, 35 patients will be recruited from seven centres over 2 years.

*Stage I*: Enrol 11 patients stop if;

1. Less than 7 of 11 patients can be planned to a dose >60 Gy EQD2
2. More than 3 of 11 patients experience grade 3+ acute radiation pneumonitis.

If three patients have experienced grade 3+ acute radiation pneumonitis before enrolment of the last patient in stage I, recruitment to stage II would only start when the last patient enrolled in stage I has reached 3 months follow-up. This would be done to ensure the absence of acute radiation pneumonitis in the fourth patient treated before proceeding to stage II.

*Stage II*: Enrol a further 24 patients, totalling 35, conclude a negative study if;

1. Less than 27 of 35 patients can be planned to a dose >60 Gy EQD2
2. More than 6 of 35 patients experience grade 3+ acute radiation pneumonitis.

There is no planned interim analysis. The study will stop after stage I if the conditions aforementioned are met.

#### Primary analysis

- Total radiation dose, proportion of patients receiving >60 Gy EQD2 to those receiving ≤60 Gy EQD2 and the reason why isotoxic IMRT≤60 Gy EQD2 will be reported.

#### Secondary analysis

- Percentage of patients who are deemed suitable to receive isotoxic IMRT, withdrawal rates, recruitment rates, incidence of toxicity, incidence of serious adverse events (SAEs), estimation of local control and estimation of overall survival (calculated from date of registration) will be reported.

### Changes to the protocol after the start of the trial

The trial details documented here are consistent with Isotoxic IMRT study protocol V.5.0 (dated: 3 December 2013). There have not been any significant changes to the protocol after the start of the study.

### End of the trial

The study will close 5 years after the last patient completes RT treatment. The chief investigator and/or the trial management group (TMG) have the right at any time to terminate the study for clinical or administrative reasons. The end of the study will be reported to the REC and Regulatory Authority (where applicable) within the required timeframes.

### QA programme

The trial is subject to a RT QA programme which is tailored to the technical requirements of lung IMRT. This programme will facilitate an audit of UK lung IMRT practice and dosimetric accuracy of delivery, as well as providing a standardised framework for implementation of lung IMRT in participating centres. The QA programme for the study is coordinated by the National Cancer Research Institute (NCRI) Radiotherapy Trials QA (RTTQA) Group. The details of the programme can be found at the RTTQA website, http://www.rttrialsqa.org.uk.

As part of the pretrial QA, sites were asked to complete the following:

- RTTQA facility questionnaire.
- Outline a benchmark case, delineating the OAR on the CT data set provided according to the study specific isotoxic IMRT atlas.
- Plan a benchmark case according to the trial protocol on the predelinated CT (OAR's, GTV, CTV and PTV).
- The first patient from each centre is reviewed before that centre recruits a second patient. If there are any problems with the first patient a prospective case review is mandated for the second patient.
- All RT plans are subject to review (retrospectively) by the Mount Vernon NCRI RTTQA Group to ensure adherence to the trials RT planning and delivery protocol.
- Finally, a site visit and complex treatment dosimetry check is carried out to review participating centres' 4D-CT and treatment verification processes and the RTTQA lung IMRT dosimetry audit.

### End of the trial

The trial will end once 35 patients have been recruited and all patients have completed 6 months follow-up or have died, whichever is sooner.

## Ethics and dissemination

### Safety reporting

Data is collected at each trial visit regarding any SAEs (as defined by GCP). All SAEs causally related to the RT treatment are reported to the MAHSC-CTU and followed until they resolve or stabilise. Acute and late radiation toxicities continue to be recorded at each follow-up visit (according to the CTCAE V.4.0 grading system).

### Trial monitoring and oversight

Formal on site data monitoring activities are performed as part of the Isotoxic IMRT study.

As this is a feasibility study data is not reviewed by an independent data monitoring committee, however, individual patient and treatment experiences are discussed at the regular TMG meetings. The TMG coordinates and manages the trial's day-to-day activities. The TMG is comprised of health professionals, a patient representative and members of the direct study team, including the principal investigators from each participating site.

### Dissemination

Data from all centres will be analysed together and published promptly. Individual participants may not publish data concerning their patients that are directly relevant to questions posed by the trial until the TMG has published its report. The TMG will form the basis of the Writing Committee and advise on the nature of publications. The trial will be publicised at regional and national conferences. The final results will be presented at scientific meetings and published in a peer-reviewed journal (authorship will be according to the journal's guidelines). In addition a lay summary of the results will be produced for interested parties for example, Cancer Research UK and BLF.

### Trial status

The first patient was registered in June 2014 and recruitment continues to stage II of the study. The study completed recruitment in March 2016. The Isotoxic IMRT trial along with three other phase I/II trials of dose-escalated sequential CTRT, will be compared with a UK standard sequential CTRT regime (55 Gy in 20 fractions) using state-of-the art RT in a randomised phase II trial. As it would be impossible to test all schedules in phase III study it was decided to proceed with a combined randomised phase II screening/“pick the winner” approach to select one schedule for further testing in a randomised phase III study.

## Footnotes

* Research & Development, Wilmslow Road, Manchester, M20 4BX, UK.
