# Correction: *Protocol for the isotoxic intensity modulated radiotherapy (IMRT) in stage III non-small cell lung cancer (NSCLC): a feasibility study*

**DOI:** 10.1136/bmjopen-2015-010457corr1

**Published:** 2016-07-18

Haslett K, Franks K, Hanna GG, *et al.* Protocol for the isotoxic intensity modulated radiotherapy (IMRT) in stage III non-small cell lung cancer (NSCLC): a feasibility study. *BMJ Open* 2016;6:e010457. This paper was published under an incorrect license. The correct license is CC BY and the following statement should be applied:

