# Supplementary material for: Protocol for the isotoxic intensity modulated radiotherapy (IMRT) in stage III non-small cell lung cancer (NSCLC): a feasibility study
Source: BMJ Open. 2016 Apr 15;6(4):e010457. doi: 10.1136/bmjopen-2015-010457 (PMC4838675; doi:10.1136/bmjopen-2015-010457)
Supplement: Supplementary appendix 1 [file bmjopen-2015-010457supp_appendix1.pdf]

**Appendix 1 – Patient Information Sheet (Version 2.0, 26th July 2013)**

**Title of the research:**

**Isotoxic Intensity Modulated Radiotherapy (IMRT) in Non-Small Cell Lung Cancer (NSCLC) – A Feasibility Study.**

**Invitation to participate in the study**

We would like to invite you to take part in our research study. Before you decide if you want to take part, we would like you to understand why the research is being done and what it will involve for you. Please read the following information carefully. One of our team will go through the information sheet with you and answer any questions you have. If you wish, discuss it with friends, relatives and your General Practitioner (GP).

**Part 1** tells you the purpose of this study and what will happen to you if you take part.
**Part 2** gives you more detailed information about the conduct of the study.

Ask us if there is anything that is not clear or if you would like more information. It is important to take time to understand this information and decide whether or not you wish to take part.

**PART 1**

**What is the purpose of this research study?**

You have been receiving chemotherapy for the treatment of a type of lung tumour called non-small cell lung cancer. Your doctor has also advised that you have a course of radiotherapy to the chest as standard treatment after your chemotherapy. Patients with your type of lung cancer have a risk of the tumour in the lung recurring or progressing after treatment.

In this study, we will investigate:

- whether giving a more targeted and individualised type of chest irradiation or radiotherapy to the lung tumour (known as Isotoxic IMRT), is practical and whether it causes side effects which can be tolerated
- whether this new method of delivering the radiotherapy can reduce the risk of the tumour in the lung recurring or progressing
- whether survival can be improved by using this new radiotherapy method

**The dose of chest irradiation will be calculated specifically to suit your body shape, the position of your lung cancer, and how close healthy tissues are to the tumour. Radiotherapy will be delivered twice a day over a maximum period of 4.5 weeks. The duration of treatment will vary individually according to the delivered dose to the tumour area.**

**What is the treatment that is being tested?**

The treatment being investigated is called isotoxic Intensity Modulated Radiotherapy (IMRT). This treatment is a new type of radiotherapy used to treat cancer.

The goal of this new type of radiotherapy is to make sure that the treatment is given more precisely to the tumour and so minimise the amount of radiation going to the surrounding healthy tissue.

In current radiotherapy treatment all patients receive the same prescription dose even though your individual disease and body shape and structure may not be suited to this. With isotoxic IMRT the radiotherapy prescription dose is individual to each patient and may therefore differ between patients. However the prescription dose for all patients in this study will be generally higher than normally given and is broken down into smaller doses given more often (twice per day). By giving a higher overall prescription dose of radiotherapy we hope to be able to control your lung cancer better.

The study will investigate whether combining individualised radiotherapy prescriptions (isotoxic) with more targeted radiotherapy (IMRT) to the tumour is practical, and whether it causes side effects which are acceptable to patients.

**Why have I been invited to take part?**

You have recently been diagnosed with non-small cell lung cancer and had a course of chemotherapy treatment. Following your chemotherapy treatment your doctor feels that you are suitable to take part in this radiotherapy study. This is a research study, and other patients similar to you are also being asked if they would be willing to take part. In total 35 patients will take part from 6 hospitals in the UK.

**Do I have to take part?**

Your participation in this research trial is entirely voluntary and you will be given sufficient time to decide whether or not you want to participate. You are free to decide at all times without giving a reason that you no longer wish to participate in the trial. Withdrawal from

the trial will not affect the standard of care you receive or your relationship with your treating doctor or the hospital staff in any way.

**What will happen to me if I take part?**

If you agree to take part, you will be asked to sign a consent form and your doctor will organise a number of tests to check if you are eligible for this study. The tests will include a physical examination, blood tests, an ECG heart trace, lung function tests and a CT scan of the chest and upper abdomen. These tests are all carried out as part of the routine tests for patients who are to be treated with radiotherapy. Some patients may also require extra tests including a pregnancy test (if you are a woman of childbearing age) and a bone scan. A CT or MR of the brain and a PET-CT scan will be requested if this has not already been done before you started chemotherapy.

If you are eligible you will have another CT scan of the chest which the doctors will use to plan your radiotherapy treatment. You will then be treated with individualised (isotoxic) and targeted (IMRT) doses of radiotherapy. Radiotherapy treatment will be delivered twice a day, Monday to Friday (excluding weekends) for a maximum period of 4.5 weeks. The radiotherapy treatment will take around 15 minutes each time and there will be a break of at least 6 hours between the two doses given each day.

If you do not live close enough to the hospital to return home during the break between the 2 radiotherapy doses you will be able to stay at the hospital. Your doctor, nurse or radiographer will be able to advise you of the facilities available to you during this time which may include, for example, a patient cafeteria, relaxation room or gardens.

After your radiotherapy treatment has finished you will have weekly out-patient follow up appointments until all your side effects have resolved. Your will then be seen at 4 weeks, and at 4, 8 and 12 months after your radiotherapy treatment and then every 6 months afterwards.

The following examinations will be performed at each follow-up visit:

- Physical evaluation & assessment of your disease status
- CT scans of the chest and abdomen (these will be carried out every 4 months from the end of treatment for 2 years)

**What are the alternatives for treatment?**

If you do not wish to take part in this study your doctor will tell you what alternatives are available in your particular situation. In most cases you will have the standard radiotherapy treatment.

**What are the possible benefits and disadvantages of taking part?**

This study may be beneficial to you. By giving a higher dose of radiotherapy we hope to be able to control your lung cancer better.. We also hope to assess whether isotoxic IMRT can improve the length of survival for lung cancer patients.

The information we get from this study may help us to treat future patients with the same disease better. Considering that the benefit of radiotherapy cannot be guaranteed we would like you to consider the possible side effects of the treatment, which are listed below:

**What are the side effects of radiotherapy to the chest?**

***Common radiotherapy side effects (occurs in more than 1 in 10 patients treated)***
Acute side effects are temporary and affect most patients; however, the severity of side effects will vary between patients. The side effects listed below will not necessarily all apply to you.

***The early side effects of radiotherapy may include:***

- Some pain in the chest in the 24 hours after the first treatment. This is usually mild and settles down fairly quickly.
- Increase in your cough and sputum (spit) which may contain a little blood. Don't worry, this is quite normal. If you are having difficulties with this during treatment, let your doctor know. The cough can sometimes worsen when treatment finishes.
- Tiredness. Tiredness related to radiotherapy varies a lot from person to person.
- Difficulty in swallowing. Inflammation of the gullet (oesophagitis) can cause discomfort when swallowing (dysphagia). Your doctor can prescribe medicines to alleviate this symptom and the hospital dietician can advise about modifications to your diet and supplements. You should concentrate on maintaining a good fluid intake.
- Shortness of breath. Inflammation of lung tissue (pneumonitis) can cause a dry cough and a degree of breathlessness during or shortly after radiotherapy. A variant of this side effect can cause troublesome breathlessness about six weeks after radiotherapy is completed. This side effect is usually treated with a course of steroid tablets.
- Skin Rash. Skin reaction can be caused by radiotherapy treatment, similar to sunburn. On rare occasions a cream may be needed.

***The late side effects of radiotherapy may include:***

- Difficulty in swallowing. Narrowing of the gullet may require a minor operation to stretch the gullet (dilatation) or in rare cases surgery. If you experience swallowing difficulties months after completion of the combined treatment further investigations (gastroscopy – tube down the gullet into the stomach) may be necessary.

- Shortness of breath. Damage to the normal lung tissue may occur from radiotherapy. This can result in shortness of breath and increased risk of infections. Radiotherapy may leave the lung with some scarring (fibrosis). This can mean that your lung does not work quite as well as it did before, and you may notice a slight increase in breathlessness.

***Rare late side effects of radiotherapy include:***

- Thinning of the ribs (following a severe cough, this can result in chest pain and/or minor rib fracture)
- Injury of the spinal cord (in extremely rare cases). An injury to the spinal cord may cause permanent difficulties in walking and loss of sensation in the lower body.
- Injury of the brachial plexus (a junction of nerves in the shoulder and neck, which control the arm and shoulder). An injury of the brachial plexus may cause numbness, weakness and tingling or pain of the shoulder, arm, or hand
- Irritation / damage to muscle or lining of the heart. Irritation/damage to the heart may cause chest pain, shortness of breath, or in rare cases heart attack. Investigations (including echocardiogram) may be necessary and you may be referred to the cardiologists for further treatment.

Every effort is made to carefully plan your treatment so as to avoid these problems. If you do have late side effects they will become noticeable 6 months or more after radiotherapy is completed and are generally permanent.

**What are the risks from the radiation?**

As part of this study, you will have more scans than with standard care; this additional radiation may slightly increase the risk of your developing cancer at a later date. However, the risk is considered to be small when compared to the possible benefits of the treatment.

**Pregnancy and birth control**

If it is possible you may conceive (i.e. you are of child bearing age), you will be asked to have a pregnancy test before starting treatment. Effective birth control with one of these methods should be used during the course of treatment.
1. Tubal ligation (informally known as getting one's "tubes tied")
2. Insertion of an intra-uterine device (IUD or coil)
3. Diaphragm with spermicidal foam/gel/film/cream/pessary
4. Condom with spermicidal foam/gel/film/cream/pessary
5. Male partner who has had a vasectomy
6. Hormonal contraceptives

If you or your partner becomes pregnant whilst you are taking part in this study, you must tell the study doctor immediately.

### 1.1.1.1.1 What happens when the research study stops?

You will continue to be followed up in the out patient clinic after the research study has finished. You may require some additional treatment in the future but this will be assessed on a case by case basis.

**What if there is a problem?**

Any complaint about the way you have been dealt with during the study or any possible harm you might suffer will be addressed. The detailed information on this is given in Part 2.

**Will my taking part in the study be kept confidential?**

Yes. We will follow ethical and legal practice and all information about you will be handled in confidence. The details are included in Part 2.

**This completes Part 1 of the information sheet.**
**If the information in Part 1 has interested you and you are considering participation, please continue to read the additional information in Part 2 before making any decision.**

## PART 2

**What if relevant new information becomes available?**

Sometimes during the course of a research project, new information becomes available about the treatment that is being studied. If this happens, your study doctor will tell you about it and discuss whether you want to or should continue in the study. If you decide not to carry on, or if your study doctor considers it to be in your best interests to withdraw you from the study, your study doctor will make arrangements for your care to continue. If you decide to continue in the study you will be asked to sign an updated consent form. If the study is stopped for any other reason, you will be told why and your continuing care will be arranged.

**What will happen if I don't want to carry on with the study?**

You can withdraw your consent at any time. This will not affect the standard of care you receive. Information collected up to your withdrawal from the study may still be used.

**What if there is a problem?**

If you have a concern about any aspect of this study, you should ask to speak to the researchers who will do their best to answer your questions. If you remain unhappy and wish to complain formally you can do this via the normal National Health Service complaints procedure.

In the event that something does go wrong and you are harmed during the research and this is due to someone's negligence then you may have grounds for a legal action for compensation against [insert institution name] but you may have to pay your legal costs.

**Will my taking part in this study be kept confidential?**

Information obtained from research will be protected and its handling will be compliant with the Data Protection Act of 1998. The doctor in charge of the study will keep your original signed consent form in a secure location. Your medical records will not hold any individual results from this study. Your unique registration number will be used to make sure you cannot be identified outside the trial. All information about you will be treated as confidential and nothing that might identify you will be revealed to any other department or organisation. Your name will not appear in any publication or report about this study.
There will be strict control of access to the files containing clinical information. Your personal information will be accessible to the study doctors and others involved in the study for the purpose of the study and your direct clinical care only. Direct access to your records may also be required by members of the independent ethics committee and/or regulatory agencies.

**Will my General Practitioner (GP) be involved?**

With your permission, your GP will be notified of your participation in this study. By signing the consent form you are agreeing to this.

**What will happen to any samples I give?**

No additional blood or tissue samples will be taken and stored specifically for this study. Any samples that are taken will be as part of your normal care. No genetic tests will be carried out.

**Will I be paid any costs and reimbursements?**

There will be no payment to you for entering this study, nor payment for undergoing investigations that are additional to your standard care.

**What will happen to the results of the research study?**

Independent experts will review the progress of the research. The results will be presented at specialist cancer meetings and published in a respected medical journal. The results will help to decide how to treat non-small cell lung cancer in the future. Studies like this are often used in cancer research.

**Who is organising and funding the research?**

This study is being organised and sponsored by The Christie NHS Foundation Trust in Manchester. The funding for this project is being provided jointly by Cancer Research UK and the British Lung Foundation. Independent researchers in the field of lung cancer have reviewed the study positively. None of the researchers involved will receive any payment for your participation in the study.

**Who has reviewed the study?**

All research in the NHS is looked at by an independent group of people, called a Research Ethics Committee, to protect your interests. This study has been reviewed and given a favourable opinion by the Greater Manchester South Research Ethics Committee.

### 1.1.1.1.2 Contact persons and further information

The research nurses and doctors listed in this section are available to answer any questions you have concerning this research study. Understand that you are free to ask any questions concerning this research study that you wish at any time.

It is important that you contact the research nurses or study doctor as soon as you experience any side effects which disrupt your daily life whether you think the treatment has caused them or not. In the event of any problem or emergency, the research nurses and doctors listed in this section may be reached during working hours (9am to 5pm):
[Insert name & contact details of research doctor/research nurse here]

If you have any concerns with your treatment and need to speak to someone outside these hours (after 5pm and before 9 am), please contact [insert institution specific hotline number].
CancerHelp UK provides general information for patients about cancer and its treatment on their website, www.cancerhelp.org.uk Cancer Research UK has cancer information nurses who provide a confidential service, Tel: 020 7061 8355 or email: cancer.info@cancer.org.uk

Macmillan Cancer Support provides support and counselling to help people living with cancer, ask Macmillan Tel: 0808 808 0000 or email www.macmillan.org.uk

UK Clinical Research Collaboration (UKCRC) publish a leaflet entitled 'Understanding Clinical Trials'. This leaflet gives you more information about medical research and looks at some questions you may want to ask. A copy may be viewed online at www.ukcrc.org or may be obtained by writing to UKCRC, 20 Park Crescent, London, W1B 1AL.

**THANK YOU FOR TAKING THE TIME TO READ THIS INFORMATION**

You will be given a copy of the information sheet and a signed consent form to keep if you decide to take part in the study.
