# Supplementary material for: Protocol for the isotoxic intensity modulated radiotherapy (IMRT) in stage III non-small cell lung cancer (NSCLC): a feasibility study
Source: BMJ Open. 2016 Apr 15;6(4):e010457. doi: 10.1136/bmjopen-2015-010457 (PMC4838675; doi:10.1136/bmjopen-2015-010457)
Supplement: Supplementary appendix 2 [file bmjopen-2015-010457supp_appendix2.pdf]

## Appendix 2 – Informed Consent Form (Version 2.0, 26th July 2013)

Patient Identification Number:

Name of Researcher: **[insert local principal investigator name]**
Title of the study: **Isotoxic Intensity Modulated Radiotherapy (IMRT) in Non-Small Cell Lung Cancer – A Feasibility Study**

**CONSENT FORM**

*Please initial in boxes*

1. I confirm that I have read and understood the information sheet Version 2, dated 26th July 2013 for the above study and have had the opportunity to ask questions.
2. I understand that my participation is voluntary and that I am free to withdraw at any time, without giving any reason, without my medical care or legal rights being affected.
3. I understand that sections of any of my medical notes may be looked at by responsible individuals from [insert institution name] and its authorised agents, by the sponsor for monitoring and audit or from Regulatory Authorities where it is relevant to my taking part in research. I give permission for these individuals to have access to my records.
4. I also understand that if I withdraw from the study early, the data collected whilst I was on the study will be retained to ensure the trial has been run in accordance with all applicable rules.
5. I understand that I will not benefit financially if this research leads to the development of a new treatment or medical test
6. I understand that my General Practitioner will be informed about my participation in this study.
7. I agree to take part in the above study.

| | | |
|---|---|---|
| Name of Patient | Date | Signature |
| Name of Person taking consent (If different from researcher) | Date | Signature |
| Researcher | Date | Signature |

1 for patient; 1 for researcher; 1 for general practitioner, 1 to be kept with hospital notes

***Thank you for taking part in this research study***
